# Cutaneous, genital and oral lichen planus: A descriptive study of 274 patients

**DOI:** 10.4317/medoral.22656

**Published:** 2018-12-24

**Authors:** Juliana Cassol-Spanemberg, Andrés Blanco-Carrión, Mª Eugenia Rodríguez-de Rivera-Campillo, Albert Estrugo-Devesa, Enric Jané-Salas, José López-López

**Affiliations:** 1 PhD. Postdoctoral Research Fellow. Specialist in Stomatology and Public Health. School of Dentistry, University of Barcelona, Spain; 2 MD, DDS, PhD. Professor of Oral Pathology, School of Dentistry, University of Santiago de Compostela, Spain; 3 MD, DDS, PhD, Dermatologist and Dentist. Professor of Oral Pathology, School of Dentistry, University of Barcelona, Spain; 4 MD, DDS, PhD. Doctor, Specialist in Stomatology. Professor of Oral Pathology, School of Dentistry, University of Barcelona, Spain / Oral Health and Masticatory System Group (Bellvitge Biomedical Research Institute) IDIBELL, University of Barcelona, Spain; 5 MD, DDS, PhD. Doctor, Specialist in Stomatology. Professor of Oral Pathology, School of Dentistry and Director of Dental Surgery, University of Barcelona. Oral Health and Masticatory System Group (Bellvitge Biomedical Research Institute) IDIBELL, University of Barcelona, Spain

## Abstract

**Background:**

Lichen planus (LP) is a chronic autoimmune disease that affects the oral mucosa as well as the skin, genital mucosa and other sites. Objective: to evaluate the correlation between oral, genital and cutaneous lichen planus, in a sample of LP patients.

**Material and Methods:**

This descriptive study reviewed 274 clinical histories of patients, who all presented histological confirmation of lichen planus verified by a pathologist, attending research centers in Barcelona.

**Results:**

A total of 40 LP patients (14.59%) presented genital lesions. Of 131 patients with cutaneous LP (47.8%), the most commonly affected zones were the body’s flexor surfaces, representing 60.1% of cases. 24% of patients (n=55) related the start of the lesions with previous stress events. Of the 131 subjects with cutaneous lesions, 19% (n=25) also presented oral lichen planus (OLP). Of the total sample, 53.6% (n=147) of patients presented oral lesions. The systemic diseases most commonly associated with this patient sample were psychological problems such as stress, anxiety and depression (48%), hypertension (27%), gastric problems (12%), and diabetes (9.7%). A family history of lichen planus was found in only 2 cases (0,72%) out of the total of 274.

**Conclusions:**

Any patient with OLP should undergo a thorough history and examination to investigate potential extraoral manifestations. The fact that 37 patients with OLP in this patient series were identified with simultaneous involvement at more than one site highlights the need for thorough evaluation and multidisciplinary approaches to this disease.

** Key words:**Oral lichen planus, extra-oral manifestations, cutaneous lichen planus, genital lichen planus.

## Introduction

Lichen Planus (LP) is a chronic inflammatory mucocutaneous disease that affects the skin, mucosa, or both. It is recurrent, of unknown etiology, and can adopt different clinical appearances depending on its time of evolution, localization, and severity. LP generally evolves with unpredictable spells of remission and intensification (1-3). The most common dermatological disease to present oral manifestations, it affects the oral mucosa with an estimated prevalence of 0.5-3%, and a female/male ratio ranging between 1.5 and 3. The age of onset is generally between 30 and 60 years and the disease also has a much-debated premalignant potential (1,4).

Exclusively oral presentation occurs in one in three patients, the oral mucosa, tongue and gums being the most common sites (5,6). Oral lichen planus (OLP) manifestations usually continue for years, alternating between periods of latency and exacerbation (7). Biopsies will confirm a clinically presumed diagnoses, and will also exclude areas showing signs of cell atypia and malignancy – manifestations that are incompatible with lichen diagnosis (4,8,9).

Histologically, the disease appears as: i) an inflammatory infiltrate in the papillary corium, which takes on a characteristic band-like distribution; ii) hyperkeratosis with ortho and/or parakeratosis; iii) vacuolating degeneration of the basal layer of the epithelium (10,11).

LP’s possible malign transformation is a highly controversial topic (9,12). Although risk is low, monitoring patients with the disease has provided evidence of its potential malignancy (13-15). Modifying some of the criteria outlined above, Warnakulasuriya *et al.* (16) included OLP as a potentially malignant disorder and most researchers recommend indefinite monitoring of OLP patients, aiming at early detection of the potential malignization.

Studies of OLP patients usually ignore extra-oral (cutaneous and/or genital) manifestations (17). Earlier research such as Eisen (1999) (18) states that genital involvement is seen in approximately 25% of men with skin involvement, whereas the frequency in women is unknown (17-19). Vulvovaginal LP can appear in isolation or in association with other manifestations at other sites. It is estimated that approximately 50% of women with OLP also present vulva affectation (17,20,21) but it also believed that this might be infra-diagnosed (22) and that in fact the incidence of affectation is higher (23-25). Meanwhile, two thirds of patients with vulva affectation also suffer vaginal and gingival affectation; this is known as vulvo-vaginal-gingival syndrome (17,26,27).

The oral form of lichen planus occurs more frequently and tends to be more resistant to treatment than the cutaneous form. In surveys of patients with oral lichen planus, the subject of extra oral manifestations has rarely been broached or if so, has focused almost exclusively on cutaneous involvement. Among the few studies that document skin lesions in patients with oral lichen planus, the reported incidence is very inconsistent, varying from 4 to 44%. Other authors affirm that approximately one third of patients presenting oral lesions also present skin lesions (3,18,28). Importantly, in addition to the skin, other affected extra-oral areas include the genital and anal mucous membranes, the scalp, and the nails (28,29).

As mentioned above, few studies have investigated the simultaneous presentation of LP in the mouth and other areas of the body. Most research has studied either oral lesions or extraoral lesions in isolation. Also, descriptive studies assessing a high number of Spanish population with lichen planus oral, cutaneous and genital is limited. In response to this scenario, the present descriptive study evaluated the correlation between oral, genital and cutaneous LP, in a sample of LP patients.

## Material and Methods

This descriptive study reviewed the clinical histories of 274 patients who all presented histological confirmation of lichen planus verified by a pathologist. The patient sample was selected from patients attending either the University of Barcelona Dental Hospital, Faculty of Dentistry (Bellvitge University Campus) (subgroup A) or the Sacrat Cor Hospital, Barcelona (subgroup B).

All patients who met the assessment criteria were included in the study. Diagnosis was determined by clinical examination and confirmed by histopathological findings characteristic of lichen planus. In this study we did not distinguish between patients with oral lichen planus and oral lichenoid lesion.

A clinical history protocol was completed for each patient registering the following variables: age and sex, clinical history, medication use, smoking and alcohol consumption, family history of LP, duration of lesions (in the mouth, wrists, feet/ankles, back, upper extremities, genitals, axillae, abdomen, nails, scalp, face, or other areas of the body) and any related situations of emotional stress clearly recognized by the patient.

The information collected was guaranteed by the confidentiality that ensures the privacy and anonymity of the subjects regarding the confidential data involved in the research. From the information obtained through the medical records, a qualitative descriptive statistical analysis, frequency tables and cross - tables were carried out, in order to verify aspects relevant to the research. The program SPSS 14.0.1 for Windows was used to analyze the data collected. *P* ≤ 0.05 was considered to be statistically significant.

## Results

The total sample included 274 patients presenting lichen planus, 93 in subgroup A (enrolled from the University of Barcelona Dental Hospital) and 181 in subgroup B (enrolled from the Sacrat Cor Hospital, Barcelona). Of the whole sample, 141 presented OLP lesions, 40 associated with genital lesions and 131 with cutaneous lesions. Table 1 shows the distribution of cases by localization. In some patients, biopsies of the different areas presenting LP manifestations were performed to confirm diagnosis.

The total sample included 208 women (75.91%) and 66 men (24.08%), showing statistically significant difference (*p*≤0.005). Age ranged between 28 and 94 years, with a mean age of 56.40 years (SD± 14.34). Age and sex did not show significant differences between the two subgroups. Of the 93 patients in subgroup A, 80.6% (n=75) were women, with a mean age of 52.82 years (SD±13.02), while of the 181 patients in subgroup B, 72.9% (n=132) were women with a mean age of 53.61 years (SD±13,24). With regard to smoking, 201 patients were non-smokers (73.4%), and of these 40 were ex-smokers (19.9%). Seventy-three patients smoked (26.6 %) (n=73), and 45 smoked over 20 cigarettes per day. Forty-four patients said that they drank alcohol (16.1%). No statistically significant differences in age and toxic habits were identified between the two subgroups.

The systemic diseases most associated with the patient sample were psychological problems such as stress, anxiety and depression (48%), hypertension (27%), gastric problems (12%) and diabetes (9.7%). This data was homogenous between the two subgroups. A family history of lichen planus was found in only two patients (0.72%) out of the total of 274, one presenting both oral and genital lesions, the other with cutaneous LP.

i) Patients with oral lesions 

Of the total sample of 274 patients, 53.6% (n=147) presented oral lesions ( Table 1). In patients with OLP, the clinical manifestation observed were: Wickham’s striae (68%), white plaques (52.3%) and erythema (36.73%). The main symptoms reported by OLP patients were pain (44.2%) and a stinging sensation (25.2%). Importantly, 36 patients (24.5%) of the 147 with oral lesions did not report any symptoms. In 116 (78.9%) of these subjects, the most affected area was the buccal mucosa, followed by the tongue (43.5%) and the gums (34.6%) ( Table 2).

**Table 1:**
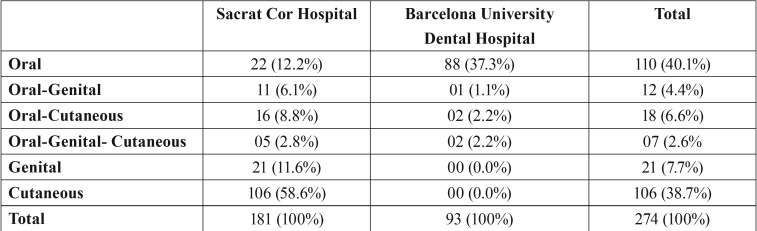
Distribution of lichen planus cases by site of origin: 147 (53.6%) presented some type of oral manifestation. 131 (47.8%) presented some type of cutaneous manifestation and 40 (14,6%) some type of genital manifestation.

**Table 2:**
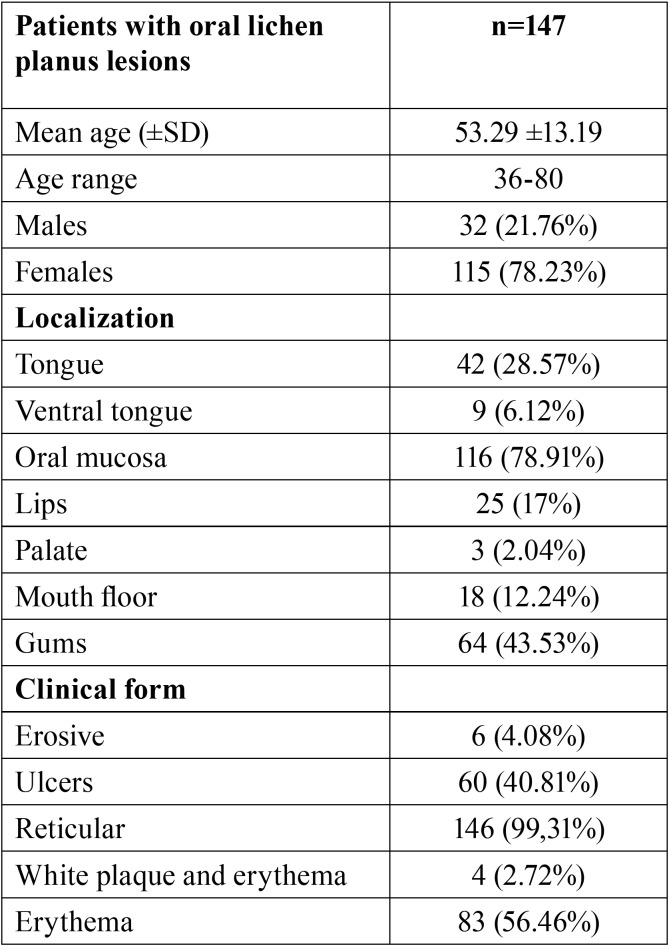
Distribution of cases of OLP by demographic data, localization and clinical type.

As for intercrisis periods, a total of 53.7% of patients with OLP reported that they suffered with oral discomfort permanently, compared with patients who claimed they suffered discomfort at intervals of one to three months.

Forty-four percent of patients with oral affectation, 12.5% of patients with genital lesions and 22% with cutaneous affectation related the onset of the lesions with prior stressful situations or events.

The most common treatment was topical corticosteroids (58.5%). An important finding was that monitoring (without any treatment) was the only approach in 39.5% of cases. The average duration of LP evolution, from the first moment of appearance, was 48 months ranging from a minimum of 3 months to a maximum of 15 years. Of the 147 patients with OLP, 2.7% (n=4) presented malignization at a previous site.

ii) Patients with cutaneous lesions

Of 131 patients presenting cutaneous lesions, the most commonly affected areas were the body’s flexor surfaces, representing 60.1% of cases. White patches associated with striae were observed in 45% of these cases. Violet papules (hyperpigmented patches) were present in 37.40% of subjects. Pruritus was the most commonly reported symptom (85.4%), followed by pain (25.9%). The mean evolution time of cutaneous lesions was 18 months, ranging from a minimum of 1 month to a maximum of 13 years. These data showed little difference between patients presenting both cutaneous and oral LP, and patients presenting cutaneous LP alone.

Of these 131 patients with cutaneous lichen planus, 19% (n=25) also presented oral lesions. Of patients presenting both cutaneous and oral lichen planus (n=25), 12% (n=3) presented cutaneous lesions prior to the appearance of oral lesions ( Table 3). The most common treatment in all cases was topical corticosteroids (85.4%).

**Table 3:**
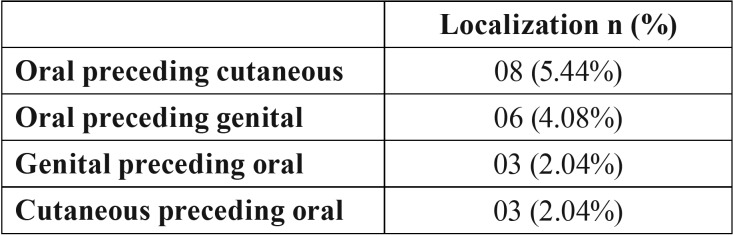
Total number and percentage of LP patients in relation to the appearance of affectation in more than one area, with one area preceding another.

iii) Patients with genital lesions 

Of the total patient sample, 14.59% (40 patients) presented genital lesions, 23 women (57.5%) and 17 men (42.5%). The most affected area in women was the vulva (55%) and in men it was the glans (42.5%). Only one case presented anal manifestation. Erythema and erosive lesions were diagnosed in 55% of genital lichen planus. As for the most common symptoms, 80% reported pruritus and 27.5% pain. Of the total number of patients with OLP, 4% (6 patients) also presented genital lesions, the genital manifestations appearing before oral manifestations, while 2% of oral lesions appeared before genital lesions.  Table 4 shows that concomitance between oral and genital lesions only occurred in two patients (1.36%).

**Table 4:**
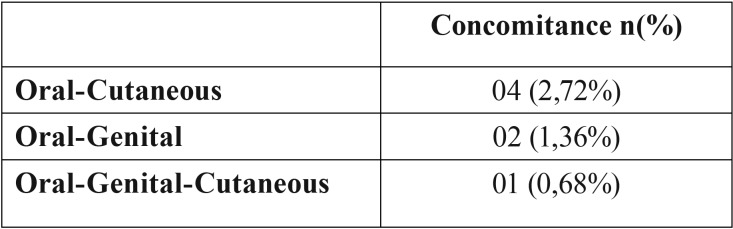
OLP cases with cutaneous and/or genital concomitance.

## Discussion

Lichen planus is a disease of the skin and mucosae; its causal factors and etiopathogenesis remain undefined. The present work evaluated a patient series with lichen planus, investigating the pathology’s oral and extraoral manifestations.

Lichen planus usually appears in subjects aged between 50 and 70 years, the majority being women, regardless of whether the disease is oral or cutaneous (17,30). The present findings coincide with other research, as 75% of patients in the present study were women with a mean age of 56 years. The average duration of discomfort reported by OLP patients was 1-3 months. Other studies report durations ranging between 3 and 12 months or even longer (up to 21 months) (31).

The results of previous studies indicate that the prevalence of smoking and alcohol consumption among OLP patients does not show statistically significant difference from the general population (32). Nevertheless, these habits should be registered in all clinical studies of patients with potentially malignant lesions. The present study analyzed smoking and alcohol consumption, finding that 27% of the sample were smokers although no significant correlation between smoking and LP was identified. This finding is comparable to other previous works, such as Gimenez-García *et al.* (31), who found that 29% of subjects with LP were smokers. Another study also concluded that smoking did not predispose individuals to developing lichen planus (33).

Although stress and depression are commonly thought to be factors for the appearance and evolution of LP, this cannot be confirmed categorically. According to the literature, the issue remains controversial, as it is difficult to determine whether or not psychic disorders are previous to the appearance of these painful chronic lesions or a consequence of the discomfort suffered. In the whole sample, the present study found that 48% of patients, 45% of whom were taking anxiolytics, reported psychological problems such as stress, anxiety and depression, without significant difference between subgroups. However, one study that included psychometric assessment, found a higher level of anxiety among OLP patients than control patients (34). In another study (31), 25% of patients clearly recognized a stressful situation previous to the appearance of cutaneous lesions and 8% suffered some depressive disorder.

Several studies note the presence of emotional problems, stress, anxiety, and/or depression as responsible for triggering the disease, and also for triggering relapses (30,34). The present results found that 41% of patients with oral lesions associated the outbreak with some stressful event, a value that dropped to 24% when the whole sample, including patients with cutaneous and genital affectation, was analyzed.

In general, the most common cutaneous affectation (whether concomitant with oral lesions or not) appears on the arms, legs or flexor surfaces and presents pruritus (29). In the present study, the distribution of lesions was similar to the distributions reported in the literature. Likewise, the severity of oral lesions did not seem to have any relation with the concomitance of cutaneous and/or genital lesions. For these patients presenting cutaneous manifestations, 60% occurred on the flexural surfaces of the body.

Oral lichen planus (OLP) can adopt different clinical forms that may be singular or combined. Wickham’s striae are the main clinical sign of OLP and are fundamental to diagnosis. In addition to the reticular form, atrophic, erosive or plaque-like lesions are also observed. In the 147 cases of OLP found in the present study, 68% presented striae, 52.3% showed white reticular lichen and 36.73% erythema. The most commonly affected zone was the oral mucosa (78.9%), followed by the tongue (43,5%) and gums (34.6%). Munde *et al.* (35) analyzed 128 patients finding that the oral mucosa was the most frequent location (88.20%); the reticular type of OLP was the most common form (83.5%) followed by erosive (15.6%) and atrophic OLP (0.78%).

As for the first appearance of lesions, the present study identified 6 patients who were aware of the presence of genital lesions but who also presented OLP of which they were unaware. Some 2% of patients presented OLP preceded by cutaneous and/or genital lesions. In this way, gynecologists or dermatologists may be the first to discover LP that is also present in the mouth, and should refer the patient to the appropriate quarter for diagnostic confirmation and treatment.

In recent years, several articles have reported a considerable increase in vulvovaginal affectation among women with OLP. These recommend systematic exploration of the genitals for women with OLP even though they may present no symptoms (20,24). In a study by Eisen (26), 19% of 399 women with OLP were found to have genital lesions and 4.6% of 174 men. Coexisting affectation of various mucosae is known as vulvo-vaginal-gingival syndrome (36,37), which is characterized by erosions and desquamation of the vulva, vagina, and gingiva (26). The present study did not identify any cases of the syndrome, although 40 patients presented lichenoid lesions in genital areas, either concomitant with other areas or not.

A family history of LP was found in only 2 patients (0,72%) out of the total of 274. Nevertheless, it is important that medical notes show the presence of a family member with the same disease, as genetic predisposition is thought to be one element involved in OLP’s etiopathogenesis (38).

For a long time, it has been suggested that patients with OLP show a greater incidence of diabetes than the general population. OLP incidence in the diabetic population is estimated to be 1.6% (39). Lopez-Jornet *et al.* (40) found type 2 diabetes in 11.5% of a sample of OLP patients. A study by Tovaru *et al.* (41) assessed 633 patients with OLP, of which 10% presented type 2 diabetes. Another study (31) observed that 10% of the OLP patients assessed were diagnosed with diabetes mellitus and 30% reported a family history of diabetes. In the present patient sample, 9.7% were diabetic which concurs with the literature cited above. However, Munde *et al.* (35), found that only 2.4% of their sample presented diabetes mellitus.

Twenty-seven per cent of patients in the present study were known to suffer hypertension, findings that exceed Lopez-Jornet *et al.* (40) and Munde *et al.* (35) who report 19,2% and 11% hypertension among their samples.

Several studies have demonstrated the premalignant potential of OLP although this remains a much-debated topic. In a cohort study by Bombeccari *et al.* (42), OLP was associated with a significant increase in the risk for oral squamous cell carcinoma (OSCC). The erosive and atrophic forms of OLP were more prevalent among patients whose lesions developed OSCC. This outcome is consistent with other reports of association between OLP and OSCC (43-46). But an earlier study of OLP patients in Northwestern Italy failed to identify any evidence that non-reticular OLP lesions are more predisposed to malignant transformation (13). In the present study, 4 patients (2.7%) presented malignization, of whom 2 were smokers and the other 2 smokers and drinkers. Kaplan *et al.* (46), in a study of 171 patients, demonstrated that the prevalence of carcinoma was 5.8% and that malignant transformation can occur in any of OLP’s clinical forms. The process of malignant transformation is still unclear (10,12,16). A recall system for OLP patients might be useful to facilitate the early diagnosis of oral cancer with the aim of reducing morbidity and mortality (9). In the present sample, the option to screen cases regularly for the sake of early detection had been taken up in 39.5% of cases.

In most cases (58.5%), the main type of treatment registered in the present study, whether oral, cutaneous, or genital LP, was the use of topical corticosteroids, a finding that concurs with the literature (35,42). Systemic corticosteroids are the first-line treatment for severe, widespread OLP and for LP involving other mucocutaneous sites resistant to topical therapies.

In many cases, the first and/or only manifestation of LP is found in the oral cavity and so it is up to the dentist to identify and manage the disease. Any patient with OLP should undergo a thorough examination to discover any other extraoral manifestations. The fact that 37 patients with OLP in this series were identified with simultaneous involvement in more than one site highlights the importance of exhaustive evaluation and the need for a multidisciplinary approach to this disease.
